# Impact of peroxisome proliferator-activated receptor-α on diabetic cardiomyopathy

**DOI:** 10.1186/s12933-020-01188-0

**Published:** 2021-01-04

**Authors:** Lin Wang, Yin Cai, Liguo Jian, Chi Wai Cheung, Liangqing Zhang, Zhengyuan Xia

**Affiliations:** 1 Department of Anesthesiology, Affiliated Hospital of Guangdong Medical University, Zhanjiang, China; 2 Department of Anaesthesiology, The University of Hong Kong, Hong Kong, SAR China; 3 Department of Health Technology and Informatics, The Hong Kong Polytechnic University, Hong Kong, SAR China; 4 Department of Cardiology, The Second Affiliated Hospital of Zhengzhou University, Zhengzhou, China

**Keywords:** Diabetic cardiomyopathy, PPARα modulator, Metformin, Glucagon-like peptide 1-receptor agonists, Sodium–glucose co-transporter type 2 inhibitors

## Abstract

The prevalence of cardiomyopathy is higher in diabetic patients than those without diabetes. Diabetic cardiomyopathy (DCM) is defined as a clinical condition of abnormal myocardial structure and performance in diabetic patients without other cardiac risk factors, such as coronary artery disease, hypertension, and significant valvular disease. Multiple molecular events contribute to the development of DCM, which include the alterations in energy metabolism (fatty acid, glucose, ketone and branched chain amino acids) and the abnormalities of subcellular components in the heart, such as impaired insulin signaling, increased oxidative stress, calcium mishandling and inflammation. There are no specific drugs in treating DCM despite of decades of basic and clinical investigations. This is, in part, due to the lack of our understanding as to how heart failure initiates and develops, especially in diabetic patients without an underlying ischemic cause. Some of the traditional anti-diabetic or lipid-lowering agents aimed at shifting the balance of cardiac metabolism from utilizing fat to glucose have been shown inadequately targeting multiple aspects of the conditions. Peroxisome proliferator-activated receptor α (PPARα), a transcription factor, plays an important role in mediating DCM-related molecular events. Pharmacological targeting of PPARα activation has been demonstrated to be one of the important strategies for patients with diabetes, metabolic syndrome, and atherosclerotic cardiovascular diseases. The aim of this review is to provide a contemporary view of PPARα in association with the underlying pathophysiological changes in DCM. We discuss the PPARα-related drugs in clinical applications and facts related to the drugs that may be considered as risky (such as fenofibrate, bezafibrate, clofibrate) or safe (pemafibrate, metformin and glucagon-like peptide 1-receptor agonists) or having the potential (sodium–glucose co-transporter 2 inhibitor) in treating DCM.

## Introduction

Diabetic cardiomyopathy (DCM) is defined as left ventricular (LV) dysfunction in diabetic patients without coronary artery disease and hypertension [1, 2]. It is usually asymptomatic in the early stages of its evolution [3]. Minimal diagnostic criteria of DCM include LV hypertrophy, interstitial fibrosis, LV diastolic dysfunction and reduced LV ejection fraction [1]. Although it remains unclear as to what initiates DCM on the molecular level, the major clinical and biochemical abnormalities in diabetes, such as hyperglycemia, systemic insulin resistance, and impaired cardiac insulin signaling, are the risk factors contributing to the pathogenesis of DCM [3, 4]. Emerging evidence highlight the importance of altered mitochondrial function as a major contributor to cardiac dysfunction in diabetes [5–7]. It has been demonstrated that in obese, insulin-resistant men, abnormal LV energy metabolism is evident prior to structural and functional pathological remodeling in the heart [8]. Clinical observations by using magnetic resonance (MR) imaging and phosphorus-31-nuclear MR spectroscopy have demonstrated that diastolic dysfunction and reduced myocardial high-energy phosphate metabolism is evident in asymptomatic normotensive male patients with well-controlled and uncomplicated type II diabetes (T2D) as compared with control subjects [9]. These findings were further demonstrated by Clarke et al. who showed that T2D patients with normal cardiac mass and function had impaired cardiac energy metabolism [10]. Mitochondrial dysfunction can occur through several mechanisms involving cardiac substrate metabolic changes, impaired cardiac insulin and glucose homeostasis [11–13], impaired cellular and mitochondrial calcium (Ca^2+^) handling [14], oxidative stress [15–17], lipotoxicity [2] and cardiac collagen deposition [18].

**Fig. 1:**
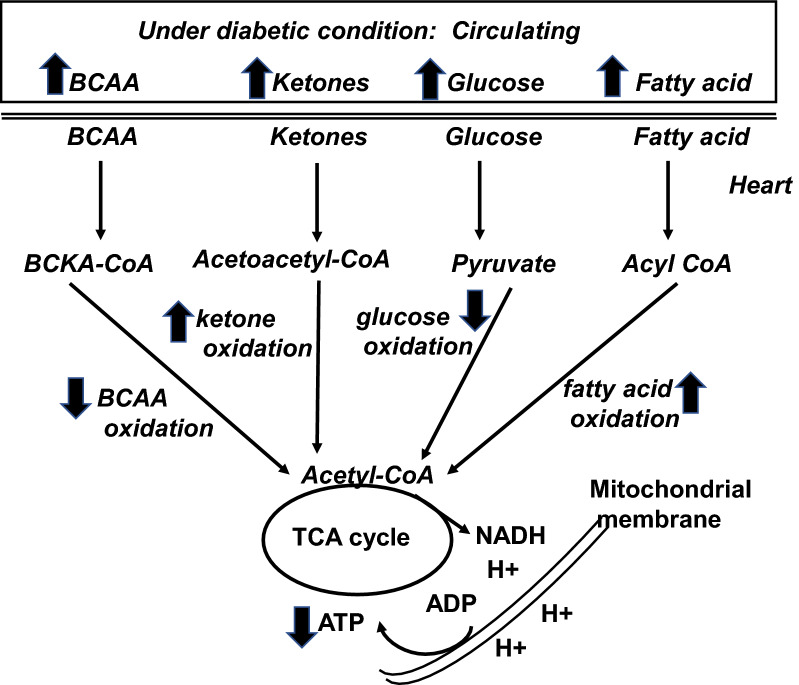
Alteration of cardiac metabolism under diabetic condition. Although circulating levels of branched chain amino acids (BCAA), ketones, glucose and fatty acids are increased under diabetic condition, their oxidation rates are not increased accordingly. Reduced BCAA and glucose oxidation, while increased ketone and fatty acid oxidation are evident in diabetic subject. Thus, reduced ATP production contributes to cardiac dysfunction

Under physiological condition, fatty acid is a major energy supplier contributing about 70% of the ATP to the working heart whereas the remaining energy relies on glucose, ketone body and branched chain amino acids (BCAA) (Fig. 1). In diabetic hearts, however, increased fatty acid uptake and decreased glucose utilization have been observed in animal models and patients [19, 20]. Meanwhile, increased utilization of ketones as an energy source is also evident in the human diabetic heart [21]. A decrease in cardiac BCAA oxidation was observed in obese mouse induced by high fat diet [13] and in heart failure patients [12]. The switch of energy substrate preference in diabetic heart occurs in parallel with higher rates of oxygen consumption and impaired oxidative phosphorylation [19]. In addition, the results obtained by using electron microscopy have demonstrated that mitochondria from diabetic patients are smaller with a reduced capacity to retain Ca^2+^ and are associated with reduced expression of mitochondrial fission protein [22]. This suggests that changes of mitochondrial ultrastructure and dynamics contribute to the development of cardiomyopathy since the impairments in mitochondrial function is associated with impaired cardiac contractility [22].

Although patients with diabetes may not all develop heart failure, clinical trials have shown that the incidence of heart failure was increased in both male and female diabetic patients when compared with age-matched individuals [23–27]. During the last decade, considerable progress has been made in DCM management by pharmacological interventions mainly through lipid-lowering therapies [28] and glycemic control strategies for the prevention of heart failure targeted at the diabetic population [29]. Peroxisome proliferator-activated receptor α (PPARα), a transcription factor which can present in a high concentration in the heart, is involved not only in regulating lipid metabolism and glucose homeostasis [30, 31], but also in Ca^2+^ handling, inflammation and oxidative stress in heart [32]. Mice with cardiac specific PPARα over-expression exhibited a similar phenotype of DCM, with LV hypertrophy, systolic dysfunction and reduced uptake of Ca^2+^ into sarcoplasmic reticulum [33]. Conversely, increases in fatty acid oxidation and uptake in diabetic hearts were significantly reduced in PPARα deficiency mice in parallel with an increase in glucose metabolism [31, 34]. Thus, modulating cardiac PPARα to prevent metabolic alterations may be a promising therapeutic strategy in DCM management.

Diastolic dysfunction, as an early sign of DCM [1], is detected in 40–75% of patients with type 1 diabetes (T1D) or T2D [35, 36] patients and in rodent diabetic models [16] in the absence of overt vascular dysfunction or atherosclerosis, suggesting a cardiac-specific response to diabetes. Excess adiposity and altered fat distribution have been shown to contribute to DCM in T1D similar to T2D [37]. However, the deleterious effects of diabetes on myocardial parameters are not all the same in patients with T1D versus T2D. For example, T1D is mostly associated with hyperglycemia, oxidative stress, myocardial fibrosis [37]. In contrast, T2D is more linked to hyperinsulinemia, insulin resistance, obesity, and cardiomyocyte hypertrophy [38]. Glycemia is a powerful promoter of heart failure in diabetic patients [39, 40], as each 1% rise in glycated hemoglobin A1C level is linked to a 30% increase in the risk of heart failure in T1D mellitus [39] and an 8% increase in risk in T2D mellitus, independent of other risk factors such as obesity, smoking, hypertension, dyslipidemia, and coronary heart disease [1, 40]. In contrast, a 1% reduction in hemoglobin A1C in T2D patients was associated with a 16% risk reduction for the development of heart failure [40]. Clearly, more studies are required to understand the differences in phenotype and underlying mechanisms for DCM in T1D and T2D.

Owing to the inherent limitations of performing mechanistic studies in humans, many of the insights into the underlying mechanisms of DCM have come from investigating rodent models. However, many of the observations in rodent models of obesity, insulin resistance, and diabetes mimic what has been seen in humans with these same pathologies. In addition, although the initial cause of impaired glucose utilization is different between T1D and T2D diabetes, ultimately they share similar downstream metabolic consequences as a result of increasing cardiomyocyte fatty acid utilization and decreased glucose utilization [1, 7]. Considering that T2D is by far the most common type of diabetes, in this review, we focus on evidence behind DCM in T2D to discuss the roles of PPARα not only in energy metabolism but also in insulin resistance, oxidative stress, inflammation, and Ca^2+^ handling regulation. We discuss the PPARα-related drugs in clinical applications. Based on the new breakthrough that some anti-diabetic drugs are associated with a lower risk of heart failure hospitalization in patients with cardiovascular disease, we also discuss PPARα-related drugs that may be risky (such as fenofibrate, bezafibrate, clofibrate) or relatively safer (pemafibrate, metformin and glucagon-like peptide 1-receptor (GLP-1R) agonists) or drugs that may have the potential (sodium–glucose co-transpoter 2 inhibitors:SGLT2i) in treating DCM.

## PPARα related mechanisms in the pathogenesis of diabetic cardiomyopathy

### Alterations of PPARα expression in DCM

A major physiological role of PPARα in the heart is to regulate the expression of target genes involved in energy metabolism *via* transactivation or transrepression through distinct mechanisms. However, abnormally increased cardiac PPARα expression has been suggested to be an important player in the development of DCM. This notion is supported by the experimental data that over-expression of PPARα resulted in the development of severe cardiomyopathy in mice [33], whereas inhibition of PPARα prevented the development of DCM [41, 42]. Likewise, mice with over-expression of PPARα on a low-fat diet also develop DCM [43]. However, clinical studies have demonstrated that the expression of PPARα is not significantly altered in the hearts of type II diabetic patients [44]. As a transcription factor, the functional expression of PPARα as reflected by its transcriptional activity is more important than its gene or protein expression. However, neither the expression profile of PPARα in relation to its activity in the context of DCM in patients, nor the co-relation of PPARα activity with cardiac function has been specifically studied.

### PPARα and mitochondrial biosynthesis in DCM

Peroxisome-proliferator-activated receptor gamma-coactivator-1α (PGC1α) has been widely accepted as a master regulator of fatty acid oxidation by modulating gene expression in the failing heart [45], and mitochondrial biogenesis in DCM [46]. Signaling of PGC1-α through activation of PPARs has been shown to control the molecules involved in mitochondrial citric acid cycle and electron transport chain [47]. On the other hand, PGC1β, which shares significant sequence homology with PGC1α [48], is also upregulated in the T2D db/db mouse heart [49], and the PGC1β/PPARα pathway has been shown to be involved in DCM through regulating cardiac metabolism [49]. This notion is further supported by the observation that knockdown of PGC1β reduced the transcriptional activity of PPARα, in parallel with an improved cardiac metabolism and cardiac dysfunction [49]. Collectively, mitochondrial dysfunction plays a pivotal role in the development of DCM, while modulating PPARα activity *via* PGC1 is a promising approach to attenuate mitochondrial dysfunction.

### PPARα and mitochondrial energy metabolism in DCM

#### Effect of PPARα on mitochondrial fatty acid and glucose oxidation in DCM

Under normal circumstances, fatty acids are the predominant energetic substrate for the heart, providing 50–70% of myocardial ATP [50]. After transport into cardiomyocytes, the majority of fatty acids are imported into mitochondria for β-oxidation, and the remaining are re-esterified into triglycerides as energy storage [50]. Cardiac PPARα is such a regulator mediating fatty acid oxidation in both neonatal heart and adult heart. The cardiac PPARα expression increases in the postnatal period [51, 52] and is responsible for regulating the expression of genes involved in fatty acid metabolism [53]. Gene expression of PPARα was decreased, in concert with reduced fatty acid oxidation in the hypertrophied newborn rabbit heart [54], while chronic stimulation of PPARα has been shown to lead to elevated fatty acid oxidation and improved cardiac function [55]. Similarly, PPARα gene expression is downregulated in the failing heart of adult mice induced by pressure overload, in parallel with a reduced fatty acid oxidation but an accumulation of triglyceride and diacylglycerol [30].

The expression of PPARα is increased in pathological conditions that accompanied with insulin resistance and in diabetes mellitus where metabolisms are impaired, suggesting its potential role in enhancing fatty acid transport and oxidation observed in diabetic hearts [33, 55]. Indeed, in diabetes, increased circulating concentrations of fatty acids activate PPARα [6], that, in turn, modulates the expression of genes involved in fatty acid uptake (such as CD36, which facilitates a major fraction of fatty acid uptake), mitochondrial transport (such as carnitine palmitoyl transferase 1), and oxidation [45]. This notion is further supported by a recent finding which shows that the abundance of the carnitine transporter OCTN2, a downstream target of PPARα, is decreased in patients with DCM [56]. There is a strong evidence from PPARα agonist and PPARα deficiency mouse models which shows that carnitine transporter OCTN2 expression contributes to tissue levels of carnitine, including that in the cardiac tissue [57]. Deficiency of carnitine, due to mutations in the carnitine transporter OCTN2 gene, is known to be associated with heart failure [58]. Of interest, the reduced expression of carnitine transporter OCTN2 is evident in patients with chronic DCM [59]. Furthermore, hearts from PPARα transgenic mice are characterized by increased fatty acid oxidation and a metabolic phenotype similar to that found in DCM [33, 60]. Taken together, these findings provide strong evidence that increased PPARα facilitates mitochondrial fatty acid metabolism in DCM.

Besides the altered fatty acid metabolism, the decrease in glucose metabolism is also evident in diabetic heart [61–63]. PPARα induces the expression of pyruvate dehydrogenase kinase 4, thereby decreasing pyruvate dehydrogenase activity leading to further suppression of glucose oxidation [55, 64]. PPARα overexpression in the mouse myocardium attenuates glucose transporter gene expression and glucose uptake [65] as well as enzymes key for glycolysis such as phosphofructokinase [66]. Thus, abnormal alteration of PPARα in response to pathological situations like diabetes and the subsequent PPAR mediated increase in fatty acid oxidation and decrease in glucose metabolism contribute to the cardiac dysfunction observed in DCM [67, 68].

#### PPARα in relation to BCAA metabolism in DCM

**Fig. 2:**
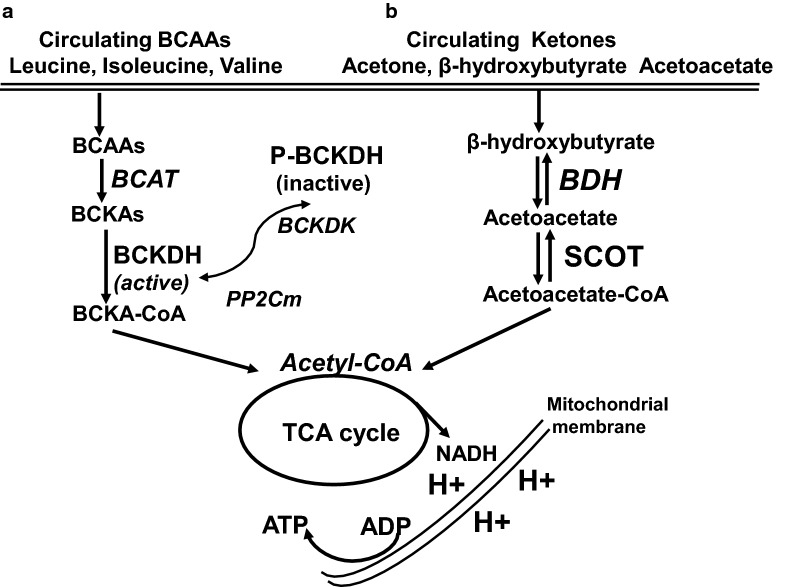
Metabolism of branched chain amino acids (BCAAs) and ketones. **a** BCAAs consist of valine, leucine and isoleucine. The metabolic homeostasis of BCAAs is controlled by a series of BCAA catabolic including branched chain aminotransferase (BCAT) and branched chain α-keto acid dehydrogenase (BCKDH). As the rate-limiting enzyme of BCAA degradation, activity of BCKDH is regulated by phosphorylation/inactivation via BCKDH-kinase (BCKD), while dephosphorylated and activated by a mitochondrion-localized protein phosphatase-2C. BCAAs are converted to acetyl CoA, which enters the tricarboxylic acid cycle (TCA) and electron transport chain (ETC) to generate ATP. **b** Ketone bodies are produced predominantly in the liver through ketogenesis, as the form of acetone, β-hydroxybutyrate and acetoacetate. The later two are the the main ketones circulating in the blood and transported to heart for oxidation. Once ketones enter the cardiomyocytes and be transported to the mitochondrial matrix, β-hydroxybutyrate and acetoacetate is oxidized into acetyl-CoA by β-hydroxybutyrate-dehydrogenase (BDH) and succinyl-CoA:3-oxoacid-CoA-transferase (SCOT). Acetyl CoA enters TCA and ETC to generate ATP

Elevated circulating level of BCAA correlates with an increasing risk of insulin resistance and T2D in human and in rodent models [69]. Reduced cardiac BCAA oxidation was observed in obese mice induced by high fat diet [13] and in heart failure patients [12]. BCAAs consist of valine, leucine and isoleucine. The metabolic homeostasis of BCAAs is controlled by a series of BCAA catabolic enzymes (Fig. 2), including branched chain aminotransferase (BCAT) and branched chain α-keto acid dehydrogenase (BCKDH) [70]. As the rate-limiting enzyme of BCAAs degradation, BCKDH is regulated by phosphorylation/inactivation via BCKDH-kinase (BCKDK), while dephosphorylated and activated by a mitochondrion-localized protein phosphatase-2Cm (PP2Cm) [70].

Although BCAAs can be highly oxidized in the heart, their contribution to cardiac ATP production only counts about 1–2% [13]. Thus, one of the important roles of BCAAs in the heart is to modulate glucose and fatty acid metabolism [71, 72]. This notion is further supported by the observation that chronic accumulation of BCAAs reduced glucose oxidation by inactivating mitochondrial pyruvate dehydrogenase [73]. Increasing cardiac BCAAs by dietary BCAA intake or genetic knockout of PP2Cm, directly contributes to the pathogenesis of a variety of cardiometabolic diseases, including heart failure [72]. Importantly, impaired BCAA oxidation as reflected by the decreased expression of BCAA metabolic enzymes has been demonstrated in patients with DCM [12].

Of interest, a most recent study shows that BCAAs accumulation due to its catabolic defects sensitizes the cardiomyocytes to ischemia/reperfusion injury through enhancing PPARα-mediated fatty acid oxidation and lipid peroxidation [74], and that chronic accumulation of BCAAs in PP2Cm global knockout mouse heart exacerbates ischemia/reperfusion-induced injury [74]. This occurs in conjunction with an enhanced glycolysis and reduced glucose oxidation *via* enhancing PPARα dependent fatty acid oxidation [74]. The molecular signaling is that BCAA or its ketone form inactivates the general control nonderepresible-2 (GCN2) and upregulates the activating transcription factor-6 (ATF6), thereby promoting PPARα transcription [74]. This is further supported by other findings showing that amino acid starvation leads to phosphorylation and activation of GCN2 [75], while ATF6 is a transcription factor essential for PPARα transcription [76]. Importantly, the ischemia/reperfusion-induced injury was attenuated in conjunction with a reduced fatty acid oxidation [74], by adenovirus-mediated PPARα silencing and by pharmacological inhibition of PPARα [74]. These results have put one step forward for understanding the association of BCAA metabolism with PPARα.

#### PPARα in relation to ketone metabolism in DCM

Ketones have long been knowing as an energy substrate to be oxidized in the heart [77]. Elevated ketone body levels have been observed in individuals with diabetes and heart failure [78]. Meanwhile, *c*ardiac ketone oxidation can increase significantly in response to changes in the arterial concentration of ketones [79] by mass action of ketone flux [80]. Increase in ketone oxidation has been observed in the failing hearts from mouse [81, 82], rat [83], and patients [56, 84].

Ketone bodies are produced predominantly in the liver through ketogenesis, during the period of fasting, in the form of acetone, β-hydroxybutyrate (β-OHB) and acetoacetate. The later two are the the main ketones circulating in the blood [80] and being transported to extrahepatic tissues for oxidation due to very low activity of β-OHB dehydrogenase in liver [85]. Once circulating ketones enter the cardiomyocytes and be transported to the mitochondrial matrix, β-OHB is oxidized into acetoacetate by β-OHB dehydrogenase (BDH) [86], after which acetoacetate together with succinyl-CoA and be converted to acetoacetyl-CoA via succinyl-CoA:3-oxoacid-CoA-transferase (SCOT), the rate limiting enzyme that is encoded by the gene *Oxct1*. Lastly, acetoacetyl-CoA is catalyzed by acetyl-CoA acetyltransferase to become acetyl-CoA, which then enter the tricarboxylic acid cycle and electron transport chain to generate ATP (Fig. 2).

Diabetes causes an elevation of circulating ketones [87]. Dysfunction of insulin coupled with glucagon release increases hepatic gluconeogenesis and ketogenesis [88]. Buildup of circulating ketones potentially results in a drop of blood pH levels that can lead to ketoacidosis, a devastating complication [88]. The increase in the level of circulating β-OHB has been observed in patients with advanced heart failure in the absence of a history of diabetes [89]. Elevation in the expression of BDH and SCOT is evident in the myocardium of patients with DCM [56], implicating an increased cardiac ketone oxidation. Mice with cardiac specific SCOT deficiency exhibit accelerated pathological ventricular remodelling in response to surgically induced pressure overload injury [90]. Thus, ketones, certainly, are the vital alternative metabolic fuel, but whether the increased utilization in the failing heart is adaptive or maladaptive remains unclear [56].

Relevant to PPARα, which is recognized as a master transcriptional regulator of ketogenesis [91], a decrease in hepatic expression of genes related to ketogenesis was observed in PPARα-deficient mice that was accompanied with aggravated steatohepatitis in mice fed a high saturated-fat diet and with the inability to maintain ketone body levels during fasting [34, 91, 92]. Β-OHB may play a key role in maintaining bio-energetic homeostasis in DCM where cardiac glucose utilization is reduced [93]. To this point, treatment with empagliflozin, a sodium-glucose co-transporter-2 antagonist, increased ketone levels, which could be a more efficient energy source in the failing myocardium of diabetic patients with heart failure [78] to compensate for the decreased myocardial glucose utilization [78]. However, the direct role of PPARα in the diabetic heart needs further studies to define.

### PPARα and abnormalities of subcellular components in DCM

An important feature of PPARα transgenic mice is the lipid accumulation in the heart [94], in which, diacylglycerol is closely associated with cardiac insulin resistance [95], while elevation of ceramide is related to cardiac dysfunction [96]. Accumulation of toxic lipids in heart is a hallmark of DCM distinct from atherosclerotic cardiovascular diseases [2], and precede the onset of diabetes and contractile dysfunction in T2D patient [97]. The increase in the level of ceramide [98] and diaceylglycerol [99] is evident in human failure heart.

PPARα confers anti-inflammatory effects mainly through inhibiting the activity of nuclear factor kappa-light-chain-enhancer of activated B cells (NFκB) [100]. NFκB has been observed in myocardial tissues from patients with heart failure as reflected by the overexpression of NFκB-regulated genes [101]. Importantly, NFκB can be activated by excessive circulating low density lipoprotein or glucose during the pathological courses of DCM [102, 103].

The association of oxidative stress with DCM has been demonstrated by elevated levels of O-linked N-acetylglucosamine (O-GlcNAc) in DCM and heart failure [1] and in animal models of diabetes [104, 105]. Excessive glucose can increase O-GlcNAcylation event, which in turn up-regulates posttranslational modification of proteins in the diabetic heart to reduce mitochondrial function and ATP production [106]. This ultimately leads to cardiac dysfunction and heart failure [106]. In supporting, mice with overexpression of antioxidant protein metallothionein are protected from developing DCM [107]. Inhibition of NFκB with pyrrolidine dithiocarbamate improved mitochondrial structural integrity in parallel with a reduced oxidative stress but increased ATP synthesis and nitric oxide bioavailability thereby restoring cardiac function in T2D [108].

Lastly, over-expression of cardiomyocyte-specific PPARα causes decreased uptake of Ca^2+^, LV hypertrophy and systolic dysfunction in the mouse heart [33]. Ca^2+^ handling is mainly controlled by the Ca^2+^ transporters, including voltage sensitive L-type Ca^2+^ channels, troponin C, Na^+^/Ca^2+^ exchanger, sarcoplasmic reticulum Ca^2+^α ATPase (SERCA2a) and the plasma membrane Ca^2+^ pump [109, 110]. Ca^2+^ mishandling has been observed not only in animal models with T1D [111, 112] or T2D [113, 114] with DCM, but also in patients with both ischemic and non-ischemic cardiomyopathy [115].

## Current therapeutic options related to PPARα for treating DCM

To date, the DCM treatment is not specifically tailored for diabetic patients with cardiac dysfunction, neither are the clinical trials [116]. The most common co-existing conditions that cause heart failure in patients with T2D are cardiovascular disease and hypertension. Therefore, antidiabetic drugs and anti-heart failure drugs have been considered in treating heart failure patients with T2D.

The most prescribed medications for diabetic patients were metformin, sulfonylureas, insulin, thiazolidinediones (Pioglitazone, Rosiglitazone). The new class of antidiabetic medications include the dipeptidyl peptidase-4 (DPP4) inhibitors (such as saxagliptin, sitagliptin, liraglutide, and alogliptin), GLP-1R agonists and SGLT2i [117]. The anti-heart failure drugs, such as angiotensin‐converting enzyme inhibitors [117], angiotensin receptor blockers [118], beta‐blockers [119] and mineralocorticoid receptor antagonists [120] have been tried in heart failure patients with T2D. However, over the years it has been found that some of the anti-diabetic drugs, such as Liraglutide [121, 122] and saxagliptin [123, 124] increase the risk for heart failure. Meanwhile, the prescribed drugs for treatment of heart failure, such as rosiglitazone [124–129] and pioglitazone [130] either showed similar effect between heart failure patients with or without diabetes [131] or increased the hospitalization risk for heart failure patients in clinical trials. In contrast, a significant breakthrough in cardiology is the finding that some anti-diabetic drugs are associated with a lower risk of heart failure hospitalization in patients with cardiovascular disease. Among which, some are PPARα-related as listed below.

*Ligands for activation of PPARα * The endogenous ligands of PPARα include fatty acids (saturated or unsaturated fatty acids [132] and its metabolites [133, 134], while the exogenous ligands are synthetic pharmaceutical agents. Current therapeutic options related to PPARα agonists include synthetic lipid-lowering drugs and glucose-lowering drugs. Of which, the former includes the conventional fibrates [135, 136] and pemafibrate, a selective PPARα modulator [41], while the later include metformin, SGLT2i and GLP-1R agonists [1].

*PPARα-related drugs that might be risky in treating DCM* The conventional fibrates, such as fenofibrate, bezafibrate, and clofibrate, are mainly excreted from the kidney [137, 138]. Their effect on antidiabetic microvascular disorders has been demonstrated in a number of large-scale clinical studies [139–141]. However, various off-target effects, such as, deterioration in liver and kidney function were evident [142, 143], indicating that their clinical efficacy is not reliable. Administration of gemfibrozil significantly reduced cardiovascular event rate (the primary endpoints of the trials), but significant drug-drug interactions between gemfibrozil and cerivastatin resulted in a very high incidence of rhabdomyolysis in patients [144]. On the other hand, the meta-analysis showed that treatment with fibrates did not significantly reduce the total mortality rate [145–147]. Thus, it was confirmed that bezafibrate [148] and fenofibrate [140] have a significant inhibitory effect on cardiovascular events. The lack of a significant mortality benefit by fibrates has led many doctors to consider them as second line drugs [143, 149].

**Fig. 3:**
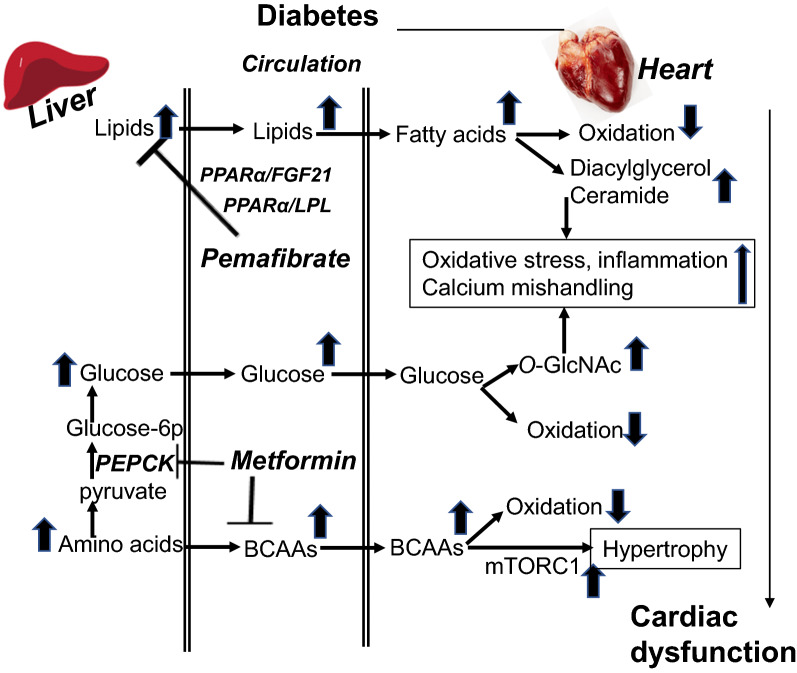
PPARα, pemafibrate, metformin and cellular abnormalities in diabetic cardiomyopathy (DCM). In diabetes, enhanced hepatic lipids, glucose and BCAA output are enhanced, resulting in lipids accumulation, increased O-GlcNAcylation and mTORC1 activation in heart. Along with the altered mitochondrial energy metabolism, these intracellular abnormalities contribute to the formation of cardiac hypertrophy and cardiac dysfunction. Pemafibrate, a novel selective PPARα modulator, is highly effective in activating PPARα than the conventional fibrates and having a better triglyceride-lowering activity through up-regulating not only hepatic fibroblast growth factor 21 (FGF21), but also lipoprotein lipase (LPL) in mice. Metformin is used in treating DCM due to its effect on reduces hepatic gluconeogenesis by inhibiting phosphoenolpyruvate carboxykinase (PEPCK). In addition, an effect of metformin in reducing circulating branched chain amino acids (BCAA) is observed in insulin-resistant mice. Further studies need to clarify if metformin may reduce cardiac hypertrophy through down-regulating BCAA-mediated mTORC1 pathway

*PPARα-related drugs that might be safe in treating DCM* Different from conventional fibrates, pemafibrate (K-877, Parmodia™), a novel selective PPARα modulator, is mainly metabolized by the liver [150, 151], and is 2500 times effective in activating PPARα than the conventional fibrates [41, 150] and having a better triglyceride-lowering activity. One of the mechanisms for lowering triglyceride is that, pemafibrate, through PPARα (Fig. 3), up-regulates not only hepatic fibroblast growth factor 21(FGF21) [152], but also lipoprotein lipase (LPL) in mice [152]. FGF21 is known to reduce secretion of very low-density lipoprotein (VLDL) [153], while LPL is known to catalyze the hydrolysis of triglyceride in VLDL and chylomicrons [154]. Its higher efficacy over fenofibrate has been confirmed by several clinical trials in Japan [155–158]. The safety profile of pemafibrate has shown no clinically adverse effects on renal or hepatic function [157, 159], and it is well tolerated over 52 weeks in T2D patients with hypertriglyceridemia [159], and in patients with impaired kidney function [160]. Importantly, pemafibrate does not show any drug interactions with various statins as demonstrated in two clinical studies [161].

With respect to cardiovascular event, pemafibrate has more potent anti-atherosclerotic effect [155–157] and glucose-lowering effect [162, 163] than the conventional fibrates in dyslipidaemic patients or hypertriglyceridemic patients with T2D. The decrease in fibrinogen expression has been demonstrated to be a predictor of reduced mortality [164], while a superior fibrinogen reducing effect of pemafibrate has been demonstrated when compared to fenofibrate in a 12-week clinical trial with dyslipidaemic patients [165]. In addition, postprandial hypertriglyceridemia is a known risk factor for cardiovascular disease due to increases in atherogenic chylomicron remnants. Pemafibrate significantly reduced postprandial triglycerides in dyslipidemic patients [163] and in diabetic patients [159, 162]. Furthermore, fasting blood glucose and insulin levels were significantly reduced by pemafibrate in hypertriglyceridemic patients with T2D relative to placebo [166]. To date, a clinical study to investigate the effect of pemafibrate on reducing cardiovascular events is on going with large-scale of T2D patients and involving multiple countries [167]. Thus, pemafibrate is expected to have a superior benefit-risk balance, and offer the potential for ameliorating diabetic microvascular complication.

*Metformin* Clinical studies have shown that metformin reduces gluconeogenesis in T2D in association with decreasing plasma dipeptidyl peptidase-4 activity [168] and increasing circulating levels of GLP-1 [169]. The molecular mechanisms mediating the actions of metformin is PPARα-dependent [170]. Of interest, transcription factor Kruppel-like factor 15 (KLF15) has been demonstrated as a molecular target in coordination with PGC1α for the glucoregulatory actions of metformin [171]. KLF15 has recently emerged as a critical transcriptional regulator of BCAA metabolism [72]. Given metformin is known to activate PGC-1α, which is a positive regulator of BCAA catabolic gene expression [172, 173], one might expect metformin to affect circulating BCAA levels. Indeed, metformin reduces circulating BCAA in insulin-resistant mice [174] via a mechanism to favour serotonergic neurotransmission in the hippocampus and promote antidepressant-like effects in mice fed a high fat diet [174].

Given that individuals with T2D have higher levels of circulating BCAA [175] and a strong link exists between dysregulated BCAA and cardiac function, one would propose that metformin treatment for diabetic patients may decrease circulating BCAA levels, thereby preventing cardiac hypertrophy via BCAA-mediated mTORC1 pathway (Fig. 3). Further investigation of the mechanisms by which KLF15 or BCAA is dysregulated in cells/tissues would provide additional insight into metformin action for the development of new antidiabetic drugs. Based on the observational studies, it has been proposed that metformin is associated with lower mortality and heart failure hospitalization rates than insulin and sulphonylureas [176]. Metformin has been suggested as first-line treatment for patients with T2D and heart failure who have preserved or moderately reduced renal function [176]. However, the effect of metformin has not been assessed in clinical trials in diabetic patients with heart failure. Thus, the statement that metformin is efficacious and safe for T2D patients with hear failure is inconclusive.

*GLP-1 receptor agonists* In the past decade, GLP-1 and its analogs have been introduced as a new class of antidiabetic medications [177]. Most recent study has demonstrated that GLP-1 agonist exendin-4 ameliorated cardiac cardiac lipotoxicity in DCM via PPARα pathway in diabetic mice [42]. GLP-1 agonists attenuate apoptosis in rat cardiomyocytes [178], while enhance nitric oxide-induced vasodilation, and facilitate glucose use in the myocardium [179, 180]. In addition, GLP-1R is highly expressed in the heart, and prominent in the therapeutics of T2D due to their efficacy in glycemia, safety, low risk of hypoglycemia and multilevel pathophysiological superiority [181] and benefits in cardiovascular disease reduction [182]. Several large placebo-controlled trials in patients with T2D and cardiovascular disease have shown that GLP‐1R agonists, such as semaglutide, have a neutral effect on reducing risk for heart failure hospitalization [183–186].

*SGLT2 inhibitors* The mechanism of SGLT2 action is through inhibiting the SGLT2 in the kidney proximal tubule leading to excretion of glucose in urea with consequent improvement in glucose control, weight reduction and decrease in blood pressure [187]. As the representative SGLT2i, empagliflozin and canagliflozin have been assessed in two randomized clinical trials for cardiovascular [188–190]. Both of which have shown a significant reduction in heart failure hospitalization [188, 190].

Recently reported evidence in cardiomyocytes have shown that SGLT2i can inhibit Na^+^/H^+^ exchanger, resulting in lowering intracellular Na^+^ and Ca^2+^ while increasing mitochondrial Ca^2+^ concentrations, ultimately improving cardiac mitochondrial function and energetics [191]. This off-target effect of SGLT2i may explain, in part, the beneficial effect of SGLT2i on heart failure [187] in relevant to the role of cardiac Na^+^/H^+^ exchanger in the pathophysiology of heart failure [192].

To date, it is unknown whether a relationship between PPARα and SGL2 inhibitor exists. A systematic review and trial-level meta-analysis using the PubMed and EMBASE databases have indicated that GLP1-R agonist and SGLT2i reduce atherosclerotic myocardial infarction, stroke, and cardiovascular death to a similar degree in patients with established atherosclerotic cardiovascular disease [193].

## Conclusions

The molecular mechanisms underlying the progression of diabetes and heart failure are closely intertwined, and the degree of clinical acceleration is greatly enhanced when the two conditions coexist as DCM. Pemafibrate, metformin and GLP-1 agonists are PPARα-related drugs that have demonstrated their efficacy and safety in reducing lipid and glucose in diabetic patients during the clinical studies. One theme common to rodent and human studies is the lack of data specific to sex differences and treatment options specific to females. Future clinical trials of heart failure treatment with these drugs that include both male and female patients with T2D would be helpful to clarify whether they can be specifically tailored for DCM patients. It is reasonable to expect that they would have a superior benefit-risk balance and offer potential for ameliorating DCM.

## Data Availability

Not applicable.

## Abbreviations

ATF6: Activating transcription factor-6
BCAA: Branched chain amino acid
BCAT: Branched chain aminotransferase
BCKDH: Branched chain α-keto acid dehydrogenase
BCKDK: BCKDH-kinase
BDH: βOHB dehydrogenase
DCM: Diabetic cardiomyopathy
DPP4: Dipeptidyl peptidase‐4
FGF21: Fibroblast growth factor 21
GCN2: General control nonderepresible-2
β-OHB: β-Hydroxybutyrate
GLP-1R: Glucagon-like peptide 1-receptor
KLF15: Kruppel-like factor 15
LPL: Lipoprotein lipase
LV: Left ventricular
MR: Magnetic resonance
NFκB: Nuclear factor kappa-light-chain-enhancer of activated B cells
*O*-GlcNAc: *O*-Linked N-acetylglucosamine
PGC1α: Peroxisome-proliferator-activated receptor gamma -coactivator-1α
PP2Cm: Protein phosphatase-2Cm
PPARα: Peroxisome proliferator-activated receptor α
SCOT: Succinyl-CoA:3-oxoacid-CoA-transferase
SERCA2a: Sarcoplasmic reticulum Ca^2^^+^α ATPase
SGLT2i: Sodium-glucose co-transpoter 2 inhibitors
T1D: Type 1 diabetes
T2D: Type II diabetes
VLDL: Very low-density lipoprotein

## References

[1] Jia G, Hill MA, Sowers JR (2018). Diabetic cardiomyopathy: an update of mechanisms contributing to this clinical entity. Circ Res.

[2] Nakamura M, Sadoshima J (2020). Cardiomyopathy in obesity, insulin resistance and diabetes. J Physiol.

[3] Jia G, DeMarco VG, Sowers JR (2016). Insulin resistance and hyperinsulinaemia in diabetic cardiomyopathy. Nat Rev Endocrinol.

[4] Cai Y, Kandula V, Kosuru R, Ye X, Irwin MG, Xia Z (2017). Decoding telomere protein Rap1: Its telomeric and nontelomeric functions and potential implications in diabetic cardiomyopathy. Cell Cycle.

[5] Chong CR, Clarke K, Levelt E (2017). Metabolic remodeling in diabetic cardiomyopathy. Cardiovasc Res.

[6] Boudina S, Abel ED (2007). Diabetic cardiomyopathy revisited. Circulation.

[7] Sung MM, Hamza SM, Dyck JR (2015). Myocardial metabolism in diabetic cardiomyopathy: potential therapeutic targets. Antioxid Redox Signal.

[8] Perseghin G, Ntali G, De Cobelli F, Lattuada G, Esposito A, Belloni E, Canu T, Costantino F, Ragogna F, Scifo P (2007). Abnormal left ventricular energy metabolism in obese men with preserved systolic and diastolic functions is associated with insulin resistance. Diabetes Care.

[9] Diamant M, Lamb HJ, Groeneveld Y, Endert EL, Smit JW, Bax JJ, Romijn JA, de Roos A, Radder JK (2003). Diastolic dysfunction is associated with altered myocardial metabolism in asymptomatic normotensive patients with well-controlled type 2 diabetes mellitus. J Am Coll Cardiol.

[10] Scheuermann-Freestone M, Madsen PL, Manners D, Blamire AM, Buckingham RE, Styles P, Radda GK, Neubauer S, Clarke K (2003). Abnormal cardiac and skeletal muscle energy metabolism in patients with type 2 diabetes. Circulation.

[11] Belke DD, Larsen TS, Gibbs EM, Severson DL (2000). Altered metabolism causes cardiac dysfunction in perfused hearts from diabetic (db/db) mice. Am J Physiol Endocrinol Metab.

[12] Uddin GM, Zhang L, Shah S, Fukushima A, Wagg CS, Gopal K, Al Batran R, Pherwani S, Ho KL, Boisvenue J (2019). Impaired branched chain amino acid oxidation contributes to cardiac insulin resistance in heart failure. Cardiovasc Diabetol.

[13] Fillmore N, Wagg CS, Zhang L, Fukushima A, Lopaschuk GD (2018). Cardiac branched-chain amino acid oxidation is reduced during insulin resistance in the heart. Am J Physiol Endocrinol Metab.

[14] Fülöp N, Mason MM, Dutta K, Wang P, Davidoff AJ, Marchase RB, Chatham JC (2007). Impact of Type 2 diabetes and aging on cardiomyocyte function and O-linked N-acetylglucosamine levels in the heart. Am J Physiol Cell Physiol.

[15] Xia Z, Kuo KH, Nagareddy PR, Wang F, Guo Z, Guo T, Jiang J, McNeill JH (2007). N-acetylcysteine attenuates PKCbeta2 overexpression and myocardial hypertrophy in streptozotocin-induced diabetic rats. Cardiovasc Res.

[16] Huynh K, Kiriazis H, Du XJ, Love JE, Jandeleit-Dahm KA, Forbes JM, McMullen JR, Ritchie RH (2012). Coenzyme Q10 attenuates diastolic dysfunction, cardiomyocyte hypertrophy and cardiac fibrosis in the db/db mouse model of type 2 diabetes. Diabetologia.

[17] Wang S, Wang C, Yan F, Wang T, He Y, Li H, Xia Z, Zhang Z (2017). N-Acetylcysteine attenuates diabetic myocardial ischemia reperfusion injury through inhibiting excessive autophagy. Mediators Inflamm.

[18] Mizushige K, Yao L, Noma T, Kiyomoto H, Yu Y, Hosomi N, Ohmori K, Matsuo H (2000). Alteration in left ventricular diastolic filling and accumulation of myocardial collagen at insulin-resistant prediabetic stage of a type II diabetic rat model. Circulation.

[19] Rijzewijk LJ, van der Meer RW, Lamb HJ, de Jong HW, Lubberink M, Romijn JA, Bax JJ, de Roos A, Twisk JW, Heine RJ (2009). Altered myocardial substrate metabolism and decreased diastolic function in nonischemic human diabetic cardiomyopathy: studies with cardiac positron emission tomography and magnetic resonance imaging. J Am Coll Cardiol.

[20] Petersen KF, Dufour S, Befroy D, Garcia R, Shulman GI (2004). Impaired mitochondrial activity in the insulin-resistant offspring of patients with type 2 diabetes. N Engl J Med.

[21] Mizuno Y, Harada E, Nakagawa H, Morikawa Y, Shono M, Kugimiya F, Yoshimura M, Yasue H (2017). The diabetic heart utilizes ketone bodies as an energy source. Metabolism.

[22] Montaigne D, Marechal X, Coisne A, Debry N, Modine T, Fayad G, Potelle C, El Arid JM, Mouton S, Sebti Y (2014). Myocardial contractile dysfunction is associated with impaired mitochondrial function and dynamics in type 2 diabetic but not in obese patients. Circulation.

[23] Aronow WS, Ahn C (1999). Incidence of heart failure in 2,737 older persons with and without diabetes mellitus. Chest.

[24] Kannel WB, Hjortland M, Castelli WP (1974). Role of diabetes in congestive heart failure: the Framingham study. Am J Cardiol.

[25] Shindler DM, Kostis JB, Yusuf S, Quinones MA, Pitt B, Stewart D, Pinkett T, Ghali JK, Wilson AC (1996). Diabetes mellitus, a predictor of morbidity and mortality in the Studies of Left Ventricular Dysfunction (SOLVD) Trials and Registry. Am J Cardiol.

[26] Rydén L, Armstrong PW, Cleland JG, Horowitz JD, Massie BM, Packer M, Poole-Wilson PA (2000). Efficacy and safety of high-dose lisinopril in chronic heart failure patients at high cardiovascular risk, including those with diabetes mellitus. Results from the ATLAS trial. Eur Heart J.

[27] Thrainsdottir IS, Aspelund T, Thorgeirsson G, Gudnason V, Hardarson T, Malmberg K, Sigurdsson G, Rydén L (2005). The association between glucose abnormalities and heart failure in the population-based Reykjavik study. Diabetes Care.

[28] Kersten S, Desvergne B, Wahli W (2000). Roles of PPARs in health and disease. Nature.

[29] Pappachan JM, Varughese GI, Sriraman R, Arunagirinathan G (2013). Diabetic cardiomyopathy: pathophysiology, diagnostic evaluation and management. World J Diabetes.

[30] Sack MN, Rader TA, Park S, Bastin J, McCune SA, Kelly DP (1996). Fatty acid oxidation enzyme gene expression is downregulated in the failing heart. Circulation.

[31] Campbell FM, Kozak R, Wagner A, Altarejos JY, Dyck JR, Belke DD, Severson DL, Kelly DP, Lopaschuk GD (2002). A role for peroxisome proliferator-activated receptor alpha (PPARalpha) in the control of cardiac malonyl-CoA levels: reduced fatty acid oxidation rates and increased glucose oxidation rates in the hearts of mice lacking PPARalpha are associated with higher concentrations of malonyl-CoA and reduced expression of malonyl-CoA decarboxylase. J Biol Chem.

[32] Lee TI, Kao YH, Chen YC, Huang JH, Hsiao FC, Chen YJ (2013). Peroxisome proliferator-activated receptors modulate cardiac dysfunction in diabetic cardiomyopathy. Diabetes Res Clin Pract.

[33] Finck BN, Lehman JJ, Leone TC, Welch MJ, Bennett MJ, Kovacs A, Han X, Gross RW, Kozak R, Lopaschuk GD (2002). The cardiac phenotype induced by PPARalpha overexpression mimics that caused by diabetes mellitus. J Clin Invest.

[34] Leone TC, Weinheimer CJ, Kelly DP (1999). A critical role for the peroxisome proliferator-activated receptor alpha (PPARalpha) in the cellular fasting response: the PPARalpha-null mouse as a model of fatty acid oxidation disorders. Proc Natl Acad Sci U S A.

[35] Boyer JK, Thanigaraj S, Schechtman KB, Pérez JE (2004). Prevalence of ventricular diastolic dysfunction in asymptomatic, normotensive patients with diabetes mellitus. Am J Cardiol.

[36] Shivalkar B, Dhondt D, Goovaerts I, Van Gaal L, Bartunek J, Van Crombrugge P, Vrints C (2006). Flow mediated dilatation and cardiac function in type 1 diabetes mellitus. Am J Cardiol.

[37] Athithan L, Gulsin GS, McCann GP, Levelt E (2019). Diabetic cardiomyopathy: Pathophysiology, theories and evidence to date. World J Diabetes.

[38] Lorenzo-Almorós A, Tuñón J, Orejas M, Cortés M, Egido J, Lorenzo Ó (2017). Diagnostic approaches for diabetic cardiomyopathy. Cardiovasc Diabetol.

[39] Lind M, Bounias I, Olsson M, Gudbjörnsdottir S, Svensson AM, Rosengren A (2011). Glycaemic control and incidence of heart failure in 20,985 patients with type 1 diabetes: an observational study. Lancet.

[40] Stratton IM, Adler AI, Neil HA, Matthews DR, Manley SE, Cull CA, Hadden D, Turner RC, Holman RR (2000). Association of glycaemia with macrovascular and microvascular complications of type 2 diabetes (UKPDS 35): prospective observational study. Bmj.

[41] Fruchart JC (2017). Pemafibrate (K-877), a novel selective peroxisome proliferator-activated receptor alpha modulator for management of atherogenic dyslipidaemia. Cardiovasc Diabetol.

[42] Wu L, Wang K, Wang W, Wen Z, Wang P, Liu L, Wang DW (2018). Glucagon-like peptide-1 ameliorates cardiac lipotoxicity in diabetic cardiomyopathy via the PPARα pathway. Aging Cell.

[43] Finck BN, Han X, Courtois M, Aimond F, Nerbonne JM, Kovacs A, Gross RW, Kelly DP (2003). A critical role for PPARalpha-mediated lipotoxicity in the pathogenesis of diabetic cardiomyopathy: modulation by dietary fat content. Proc Natl Acad Sci U S A.

[44] Razeghi P, Young ME, Cockrill TC, Frazier OH, Taegtmeyer H (2002). Downregulation of myocardial myocyte enhancer factor 2C and myocyte enhancer factor 2C-regulated gene expression in diabetic patients with nonischemic heart failure. Circulation.

[45] Riehle C, Abel ED (2012). PGC-1 proteins and heart failure. Trends Cardiovasc Med.

[46] Waldman M, Cohen K, Yadin D, Nudelman V, Gorfil D, Laniado-Schwartzman M, Kornwoski R, Aravot D, Abraham NG, Arad M (2018). Regulation of diabetic cardiomyopathy by caloric restriction is mediated by intracellular signaling pathways involving ‘SIRT1 and PGC-1α’. Cardiovasc Diabetol.

[47] Duncan JG, Fong JL, Medeiros DM, Finck BN, Kelly DP (2007). Insulin-resistant heart exhibits a mitochondrial biogenic response driven by the peroxisome proliferator-activated receptor-alpha/PGC-1alpha gene regulatory pathway. Circulation.

[48] Rowe GC, Jiang A, Arany Z (2010). PGC-1 coactivators in cardiac development and disease. Circ Res.

[49] Yin Z, Zhao Y, He M, Li H, Fan J, Nie X, Yan M, Chen C, Wang DW (2019). MiR-30c/PGC-1β protects against diabetic cardiomyopathy via PPARα. Cardiovasc Diabetol.

[50] Lopaschuk GD, Ussher JR, Folmes CD, Jaswal JS, Stanley WC (2010). Myocardial fatty acid metabolism in health and disease. Physiol Rev.

[51] Panadero M, Herrera E, Bocos C (2000). Peroxisome proliferator-activated receptor-alpha expression in rat liver during postnatal development. Biochimie.

[52] Steinmetz M, Quentin T, Poppe A, Paul T, Jux C (2005). Changes in expression levels of genes involved in fatty acid metabolism: upregulation of all three members of the PPAR family (alpha, gamma, delta) and the newly described adiponectin receptor 2, but not adiponectin receptor 1 during neonatal cardiac development of the rat. Basic Res Cardiol.

[53] Muoio DM, Way JM, Tanner CJ, Winegar DA, Kliewer SA, Houmard JA, Kraus WE, Dohm GL (2002). Peroxisome proliferator-activated receptor-alpha regulates fatty acid utilization in primary human skeletal muscle cells. Diabetes.

[54] Lam VH, Zhang L, Huqi A, Fukushima A, Tanner BA, Onay-Besikci A, Keung W, Kantor PF, Jaswal JS, Rebeyka IM (2015). Activating PPARα prevents post-ischemic contractile dysfunction in hypertrophied neonatal hearts. Circ Res.

[55] Buchanan J, Mazumder PK, Hu P, Chakrabarti G, Roberts MW, Yun UJ, Cooksey RC, Litwin SE, Abel ED (2005). Reduced cardiac efficiency and altered substrate metabolism precedes the onset of hyperglycemia and contractile dysfunction in two mouse models of insulin resistance and obesity. Endocrinology.

[56] Bedi KC, Snyder NW, Brandimarto J, Aziz M, Mesaros C, Worth AJ, Wang LL, Javaheri A, Blair IA, Margulies KB (2016). Evidence for Intramyocardial Disruption of Lipid Metabolism and Increased Myocardial Ketone Utilization in Advanced Human Heart Failure. Circulation.

[57] van Vlies N, Ferdinandusse S, Turkenburg M, Wanders RJ, Vaz FM (2007). PPAR alpha-activation results in enhanced carnitine biosynthesis and OCTN2-mediated hepatic carnitine accumulation. Biochim Biophys Acta.

[58] Wang Y, Ye J, Ganapathy V, Longo N (1999). Mutations in the organic cation/carnitine transporter OCTN2 in primary carnitine deficiency. Proc Natl Acad Sci U S A.

[59] Grube M, Ameling S, Noutsias M, Köck K, Triebel I, Bonitz K, Meissner K, Jedlitschky G, Herda LR, Reinthaler M (2011). Selective regulation of cardiac organic cation transporter novel type 2 (OCTN2) in dilated cardiomyopathy. Am J Pathol.

[60] Hafstad AD, Khalid AM, Hagve M, Lund T, Larsen TS, Severson DL, Clarke K, Berge RK, Aasum E (2009). Cardiac peroxisome proliferator-activated receptor-alpha activation causes increased fatty acid oxidation, reducing efficiency and post-ischaemic functional loss. Cardiovasc Res.

[61] Ussher JR, Koves TR, Jaswal JS, Zhang L, Ilkayeva O, Dyck JR, Muoio DM, Lopaschuk GD (2009). Insulin-stimulated cardiac glucose oxidation is increased in high-fat diet-induced obese mice lacking malonyl CoA decarboxylase. Diabetes.

[62] Carley AN, Severson DL (2005). Fatty acid metabolism is enhanced in type 2 diabetic hearts. Biochim Biophys Acta.

[63] Stanley WC, Lopaschuk GD, McCormack JG (1997). Regulation of energy substrate metabolism in the diabetic heart. Cardiovasc Res.

[64] Wright JJ, Kim J, Buchanan J, Boudina S, Sena S, Bakirtzi K, Ilkun O, Theobald HA, Cooksey RC, Kandror KV (2009). Mechanisms for increased myocardial fatty acid utilization following short-term high-fat feeding. Cardiovasc Res.

[65] Finck BN (2006). Effects of PPARalpha on cardiac glucose metabolism: a transcriptional equivalent of the glucose-fatty acid cycle?. Expert Rev Cardiovasc Ther.

[66] Goldbeter A, Lefever R (1972). Dissipative structures for an allosteric model. Application to glycolytic oscillations. Biophys J.

[67] How OJ, Larsen TS, Hafstad AD, Khalid A, Myhre ES, Murray AJ, Boardman NT, Cole M, Clarke K, Severson DL (2007). Rosiglitazone treatment improves cardiac efficiency in hearts from diabetic mice. Arch Physiol Biochem.

[68] Sharma V, Dhillon P, Wambolt R, Parsons H, Brownsey R, Allard MF, McNeill JH (2008). Metoprolol improves cardiac function and modulates cardiac metabolism in the streptozotocin-diabetic rat. Am J Physiol Heart Circ Physiol.

[69] Giesbertz P, Daniel H (2016). Branched-chain amino acids as biomarkers in diabetes. Curr Opin Clin Nutr Metab Care.

[70] Huang Y, Zhou M, Sun H, Wang Y (2011). Branched-chain amino acid metabolism in heart disease: an epiphenomenon or a real culprit?. Cardiovasc Res.

[71] Jang C, Oh SF, Wada S, Rowe GC, Liu L, Chan MC, Rhee J, Hoshino A, Kim B, Ibrahim A (2016). A branched-chain amino acid metabolite drives vascular fatty acid transport and causes insulin resistance. Nat Med.

[72] Sun H, Olson KC, Gao C, Prosdocimo DA, Zhou M, Wang Z, Jeyaraj D, Youn JY, Ren S, Liu Y (2016). Catabolic defect of branched-chain amino acids promotes heart failure. Circulation.

[73] Li T, Zhang Z, Kolwicz SC, Abell L, Roe ND, Kim M, Zhou B, Cao Y, Ritterhoff J, Gu H (2017). Defective branched-chain amino acid catabolism disrupts glucose metabolism and sensitizes the heart to ischemia-reperfusion injury. Cell Metab.

[74] Li Y, Xiong Z, Yan W, Gao E, Cheng H, Wu G, Liu Y, Zhang L, Li C, Wang S (2020). Branched chain amino acids exacerbate myocardial ischemia/reperfusion vulnerability via enhancing GCN2/ATF6/PPAR-α pathway-dependent fatty acid oxidation. Theranostics.

[75] Gallinetti J, Harputlugil E, Mitchell JR (2013). Amino acid sensing in dietary-restriction-mediated longevity: roles of signal-transducing kinases GCN2 and TOR. Biochem J.

[76] Chen X, Zhang F, Gong Q, Cui A, Zhuo S, Hu Z, Han Y, Gao J, Sun Y, Liu Z (2016). Hepatic ATF6 Increases Fatty Acid Oxidation to Attenuate Hepatic Steatosis in Mice Through Peroxisome Proliferator-Activated Receptor α. Diabetes.

[77] Bing RJ, Siegel A, Ungar I, Gilbert M (1954). Metabolism of the human heart. II. Studies on fat, ketone and amino acid metabolism. Am J Med.

[78] Mudaliar S, Alloju S, Henry RR (2016). Can a shift in fuel energetics explain the beneficial cardiorenal outcomes in the EMPA-REG OUTCOME Study? a unifying hypothesis. Diabetes Care.

[79] Rudolph W, Maas D, Richter J, Hasinger F, Hofmann H, Dohrn P (1965). On the significance of acetoacetate and beta-hydroxybutyrate in human myocardial metabolism. Klin Wochenschr.

[80] Puchalska P, Crawford PA (2017). Multi-dimensional roles of ketone bodies in fuel metabolism, signaling, and therapeutics. Cell Metab.

[81] Ho KL, Zhang L, Wagg C, Al Batran R, Gopal K, Levasseur J, Leone T, Dyck JRB, Ussher JR, Muoio DM (2019). Increased ketone body oxidation provides additional energy for the failing heart without improving cardiac efficiency. Cardiovasc Res.

[82] Aubert G, Martin OJ, Horton JL, Lai L, Vega RB, Leone TC, Koves T, Gardell SJ, Krüger M, Hoppel CL (2016). The failing heart relies on ketone bodies as a fuel. Circulation.

[83] Murray AJ, Knight NS, Cole MA, Cochlin LE, Carter E, Tchabanenko K, Pichulik T, Gulston MK, Atherton HJ, Schroeder MA (2016). Novel ketone diet enhances physical and cognitive performance. Faseb j.

[84] Du Z, Shen A, Huang Y, Su L, Lai W, Wang P, Xie Z, Xie Z, Zeng Q, Ren H (2014). 1H-NMR-based metabolic analysis of human serum reveals novel markers of myocardial energy expenditure in heart failure patients. PLoS One.

[85] Nielsen NC, Fleischer S (1969). Beta-hydroxybutyrate dehydrogenase: lack in ruminant liver mitochondria. Science.

[86] Lehninger AL, Sudduth HC, Wise JB (1960). D-beta-Hydroxybutyric dehydrogenase of muitochondria. J Biol Chem.

[87] Hall SE, Wastney ME, Bolton TM, Braaten JT, Berman M (1984). Ketone body kinetics in humans: the effects of insulin-dependent diabetes, obesity, and starvation. J Lipid Res.

[88] Wolfsdorf J, Glaser N, Sperling MA (2006). Diabetic ketoacidosis in infants, children, and adolescents: a consensus statement from the American Diabetes Association. Diabetes Care.

[89] Lommi J, Kupari M, Koskinen P, Näveri H, Leinonen H, Pulkki K, Härkönen M (1996). Blood ketone bodies in congestive heart failure. J Am Coll Cardiol.

[90] Schugar RC, Moll AR, André d’Avignon D, Weinheimer CJ, Kovacs A, Crawford PA (2014). Cardiomyocyte-specific deficiency of ketone body metabolism promotes accelerated pathological remodeling. Mol Metab.

[91] Kersten S, Seydoux J, Peters JM, Gonzalez FJ, Desvergne B, Wahli W (1999). Peroxisome proliferator-activated receptor alpha mediates the adaptive response to fasting. J Clin Invest.

[92] Patsouris D, Reddy JK, Müller M, Kersten S (2006). Peroxisome proliferator-activated receptor alpha mediates the effects of high-fat diet on hepatic gene expression. Endocrinology.

[93] Pawlak M, Baugé E, Lalloyer F, Lefebvre P, Staels B (2015). Ketone body therapy protects from lipotoxicity and acute liver failure upon pparα deficiency. Mol Endocrinol.

[94] Park SY, Cho YR, Finck BN, Kim HJ, Higashimori T, Hong EG, Lee MK, Danton C, Deshmukh S, Cline GW (2005). Cardiac-specific overexpression of peroxisome proliferator-activated receptor-alpha causes insulin resistance in heart and liver. Diabetes.

[95] Zhang L, Ussher JR, Oka T, Cadete VJ, Wagg C, Lopaschuk GD (2011). Cardiac diacylglycerol accumulation in high fat-fed mice is associated with impaired insulin-stimulated glucose oxidation. Cardiovasc Res.

[96] Park TS, Hu Y, Noh HL, Drosatos K, Okajima K, Buchanan J, Tuinei J, Homma S, Jiang XC, Abel ED (2008). Ceramide is a cardiotoxin in lipotoxic cardiomyopathy. J Lipid Res.

[97] McGavock JM, Lingvay I, Zib I, Tillery T, Salas N, Unger R, Levine BD, Raskin P, Victor RG, Szczepaniak LS (2007). Cardiac steatosis in diabetes mellitus: a 1H-magnetic resonance spectroscopy study. Circulation.

[98] Ji R, Akashi H, Drosatos K, Liao X, Jiang H, Kennel PJ, Brunjes DL, Castillero E, Zhang X, Deng LY (2017). Increased de novo ceramide synthesis and accumulation in failing myocardium. JCI Insight..

[99] Chokshi A, Drosatos K, Cheema FH, Ji R, Khawaja T, Yu S, Kato T, Khan R, Takayama H, Knöll R (2012). Ventricular assist device implantation corrects myocardial lipotoxicity, reverses insulin resistance, and normalizes cardiac metabolism in patients with advanced heart failure. Circulation.

[100] Lorenzo O, Picatoste B, Ares-Carrasco S, Ramírez E, Egido J, Tuñón J (2011). Potential role of nuclear factor κB in diabetic cardiomyopathy. Mediators Inflamm.

[101] Jones WK, Brown M, Ren X, He S, McGuinness M (2003). NF-kappaB as an integrator of diverse signaling pathways: the heart of myocardial signaling?. Cardiovasc Toxicol.

[102] Fuentes-Antrás J, Ioan AM, Tuñón J, Egido J, Lorenzo O (2014). Activation of toll-like receptors and inflammasome complexes in the diabetic cardiomyopathy-associated inflammation. Int J Endocrinol.

[103] Mazière C, Mazière JC (2009). Activation of transcription factors and gene expression by oxidized low-density lipoprotein. Free Radic Biol Med.

[104] Yan D, Cai Y, Luo J, Liu J, Li X, Ying F, Xie X, Xu A, Ma X, Xia Z (2020). FOXO1 contributes to diabetic cardiomyopathy via inducing imbalanced oxidative metabolism in type 1 diabetes. J Cell Mol Med.

[105] Luo J, Yan D, Li S, Liu S, Zeng F, Cheung CW, Liu H, Irwin MG, Huang H, Xia Z (2020). Allopurinol reduces oxidative stress and activates Nrf2/p62 to attenuate diabetic cardiomyopathy in rats. J Cell Mol Med.

[106] Dassanayaka S, Jones SP (2014). O-GlcNAc and the cardiovascular system. Pharmacol Ther.

[107] Wang J, Song Y, Elsherif L, Song Z, Zhou G, Prabhu SD, Saari JT, Cai L (2006). Cardiac metallothionein induction plays the major role in the prevention of diabetic cardiomyopathy by zinc supplementation. Circulation.

[108] Mariappan N, Elks CM, Sriramula S, Guggilam A, Liu Z, Borkhsenious O, Francis J (2010). NF-kappaB-induced oxidative stress contributes to mitochondrial and cardiac dysfunction in type II diabetes. Cardiovasc Res.

[109] Liu W, Chen P, Deng J, Lv J, Liu J (2017). Resveratrol and polydatin as modulators of Ca(2+) mobilization in the cardiovascular system. Ann N Y Acad Sci.

[110] Kanaporis G, Blatter LA (2017). Membrane potential determines calcium alternans through modulation of SR Ca(2+) load and L-type Ca(2+) current. J Mol Cell Cardiol.

[111] Ye G, Metreveli NS, Donthi RV, Xia S, Xu M, Carlson EC, Epstein PN (2004). Catalase protects cardiomyocyte function in models of type 1 and type 2 diabetes. Diabetes.

[112] Ye G, Metreveli NS, Ren J, Epstein PN (2003). Metallothionein prevents diabetes-induced deficits in cardiomyocytes by inhibiting reactive oxygen species production. Diabetes.

[113] Van den Bergh A, Vanderper A, Vangheluwe P, Desjardins F, Nevelsteen I, Verreth W, Wuytack F, Holvoet P, Flameng W, Balligand JL (2008). Dyslipidaemia in type II diabetic mice does not aggravate contractile impairment but increases ventricular stiffness. Cardiovasc Res.

[114] Belke DD, Swanson EA, Dillmann WH (2004). Decreased sarcoplasmic reticulum activity and contractility in diabetic db/db mouse heart. Diabetes.

[115] Piacentino V, Weber CR, Chen X, Weisser-Thomas J, Margulies KB, Bers DM, Houser SR (2003). Cellular basis of abnormal calcium transients of failing human ventricular myocytes. Circ Res.

[116] Ponikowski P, Voors AA, Anker SD, Bueno H, Cleland JGF, Coats AJS, Falk V, González-Juanatey JR, Harjola VP, Jankowska EA (2016). 2016 ESC Guidelines for the diagnosis and treatment of acute and chronic heart failure: The Task Force for the diagnosis and treatment of acute and chronic heart failure of the European Society of Cardiology (ESC)Developed with the special contribution of the Heart Failure Association (HFA) of the ESC. Eur Heart J.

[117] Cavender MA, Steg PG, Smith SC, Eagle K, Ohman EM, Goto S, Kuder J, Im K, Wilson PW, Bhatt DL (2015). Impact of diabetes mellitus on hospitalization for heart failure, cardiovascular events, and death: outcomes at 4 years from the reduction of atherothrombosis for continued health (REACH) Registry. Circulation.

[118] Maggioni AP, Anand I, Gottlieb SO, Latini R, Tognoni G, Cohn JN (2002). Effects of valsartan on morbidity and mortality in patients with heart failure not receiving angiotensin-converting enzyme inhibitors. J Am Coll Cardiol.

[119] Haas SJ, Vos T, Gilbert RE, Krum H (2003). Are beta-blockers as efficacious in patients with diabetes mellitus as in patients without diabetes mellitus who have chronic heart failure? A meta-analysis of large-scale clinical trials. Am Heart J.

[120] Martinson NA, Barnes GL, Moulton LH, Msandiwa R, Hausler H, Ram M, McIntyre JA, Gray GE, Chaisson RE (2011). New regimens to prevent tuberculosis in adults with HIV infection. N Engl J Med.

[121] Jorsal A, Kistorp C, Holmager P, Tougaard RS, Nielsen R, Hänselmann A, Nilsson B, Møller JE, Hjort J, Rasmussen J (2017). Effect of liraglutide, a glucagon-like peptide-1 analogue, on left ventricular function in stable chronic heart failure patients with and without diabetes (LIVE)-a multicentre, double-blind, randomised, placebo-controlled trial. Eur J Heart Fail.

[122] Margulies KB, Hernandez AF, Redfield MM, Givertz MM, Oliveira GH, Cole R, Mann DL, Whellan DJ, Kiernan MS, Felker GM (2016). Effects of liraglutide on clinical stability among patients with advanced heart failure and reduced ejection fraction: a randomized clinical trial. Jama.

[123] Scirica BM, Braunwald E, Raz I, Cavender MA, Morrow DA, Jarolim P, Udell JA, Mosenzon O, Im K, Umez-Eronini AA (2014). Heart failure, saxagliptin, and diabetes mellitus: observations from the SAVOR-TIMI 53 randomized trial. Circulation.

[124] Scirica BM, Bhatt DL, Braunwald E, Steg PG, Davidson J, Hirshberg B, Ohman P, Frederich R, Wiviott SD, Hoffman EB (2013). Saxagliptin and cardiovascular outcomes in patients with type 2 diabetes mellitus. N Engl J Med.

[125] Nissen SE, Wolski K (2007). Effect of rosiglitazone on the risk of myocardial infarction and death from cardiovascular causes. N Engl J Med.

[126] Nissen SE, Wolski K (2010). Rosiglitazone revisited: an updated meta-analysis of risk for myocardial infarction and cardiovascular mortality. Arch Intern Med.

[127] Graham DJ, Ouellet-Hellstrom R, MaCurdy TE, Ali F, Sholley C, Worrall C, Kelman JA (2010). Risk of acute myocardial infarction, stroke, heart failure, and death in elderly Medicare patients treated with rosiglitazone or pioglitazone. Jama.

[128] Bach RG, Brooks MM, Lombardero M, Genuth S, Donner TW, Garber A, Kennedy L, Monrad ES, Pop-Busui R, Kelsey SF (2013). Rosiglitazone and outcomes for patients with diabetes mellitus and coronary artery disease in the Bypass Angioplasty Revascularization Investigation 2 Diabetes (BARI 2D) trial. Circulation.

[129] Komajda M, McMurray JJ, Beck-Nielsen H, Gomis R, Hanefeld M, Pocock SJ, Curtis PS, Jones NP, Home PD (2010). Heart failure events with rosiglitazone in type 2 diabetes: data from the RECORD clinical trial. Eur Heart J.

[130] Lago RM, Singh PP, Nesto RW (2007). Congestive heart failure and cardiovascular death in patients with prediabetes and type 2 diabetes given thiazolidinediones: a meta-analysis of randomised clinical trials. Lancet.

[131] Seferović PM, Petrie MC, Filippatos GS, Anker SD, Rosano G, Bauersachs J, Paulus WJ, Komajda M, Cosentino F, de Boer RA (2018). Type 2 diabetes mellitus and heart failure: a position statement from the Heart Failure Association of the European Society of Cardiology. Eur J Heart Fail.

[132] Ricote M, Valledor AF, Glass CK (2004). Decoding transcriptional programs regulated by PPARs and LXRs in the macrophage: effects on lipid homeostasis, inflammation, and atherosclerosis. Arterioscler Thromb Vasc Biol.

[133] Guan Y, Zhang Y, Schneider A, Davis L, Breyer RM, Breyer MD (2001). Peroxisome proliferator-activated receptor-gamma activity is associated with renal microvasculature. Am J Physiol Renal Physiol.

[134] Cowart LA, Wei S, Hsu MH, Johnson EF, Krishna MU, Falck JR, Capdevila JH (2002). The CYP4A isoforms hydroxylate epoxyeicosatrienoic acids to form high affinity peroxisome proliferator-activated receptor ligands. J Biol Chem.

[135] Staels B, Dallongeville J, Auwerx J, Schoonjans K, Leitersdorf E, Fruchart JC (1998). Mechanism of action of fibrates on lipid and lipoprotein metabolism. Circulation.

[136] Jones AB (2001). Peroxisome proliferator-activated receptor (PPAR) modulators: diabetes and beyond. Med Res Rev.

[137] Ncube V, Starkey B, Wang T (2012). Effect of fenofibrate treatment for hyperlipidaemia on serum creatinine and cystatin C. Ann Clin Biochem.

[138] Mazidi M, Kengne AP, Mikhailidis DP, Cicero AF, Banach M (2018). Effects of selected dietary constituents on high-sensitivity C-reactive protein levels in U.S. adults. Ann Med.

[139] Hiukka A, Maranghi M, Matikainen N, Taskinen MR (2010). PPARalpha: an emerging therapeutic target in diabetic microvascular damage. Nat Rev Endocrinol.

[140] Keech A, Simes RJ, Barter P, Best J, Scott R, Taskinen MR, Forder P, Pillai A, Davis T, Glasziou P (2005). Effects of long-term fenofibrate therapy on cardiovascular events in 9795 people with type 2 diabetes mellitus (the FIELD study): randomised controlled trial. Lancet.

[141] Keech AC, Mitchell P, Summanen PA, O’Day J, Davis TM, Moffitt MS, Taskinen MR, Simes RJ, Tse D, Williamson E (2007). Effect of fenofibrate on the need for laser treatment for diabetic retinopathy (FIELD study): a randomised controlled trial. Lancet.

[142] Frick MH, Elo O, Haapa K, Heinonen OP, Heinsalmi P, Helo P, Huttunen JK, Kaitaniemi P, Koskinen P, Manninen V (1987). Helsinki Heart Study: primary-prevention trial with gemfibrozil in middle-aged men with dyslipidemia. Safety of treatment, changes in risk factors, and incidence of coronary heart disease. N Engl J Med.

[143] Rubins HB, Robins SJ, Collins D, Fye CL, Anderson JW, Elam MB, Faas FH, Linares E, Schaefer EJ, Schectman G (1999). Gemfibrozil for the secondary prevention of coronary heart disease in men with low levels of high-density lipoprotein cholesterol. Veterans Affairs High-Density Lipoprotein Cholesterol Intervention Trial Study Group. N Engl J Med.

[144] Jacobson TA (2009). Myopathy with statin-fibrate combination therapy: clinical considerations. Nat Rev Endocrinol.

[145] Jun M, Foote C, Lv J, Neal B, Patel A, Nicholls SJ, Grobbee DE, Cass A, Chalmers J, Perkovic V (2010). Effects of fibrates on cardiovascular outcomes: a systematic review and meta-analysis. Lancet.

[146] Sacks FM, Carey VJ, Fruchart JC (2010). Combination lipid therapy in type 2 diabetes. N Engl J Med.

[147] Saely CH, Rein P, Drexel H (2010). Combination lipid therapy in type 2 diabetes. N Engl J Med.

[148] Secondary prevention by (2000). raising HDL cholesterol and reducing triglycerides in patients with coronary artery disease. Circulation.

[149] Geiger G, Lettvin JY (1987). Peripheral vision in persons with dyslexia. N Engl J Med.

[150] Yamashita S, Masuda D, Matsuzawa Y (2020). Pemafibrate, a new selective PPARα modulator: drug concept and its clinical applications for dyslipidemia and metabolic diseases. Curr Atheroscler Rep.

[151] Fruchart JC, Santos RD, Aguilar-Salinas C, Aikawa M, Al Rasadi K, Amarenco P, Barter PJ, Ceska R, Corsini A, Després JP (2019). The selective peroxisome proliferator-activated receptor alpha modulator (SPPARMα) paradigm: conceptual framework and therapeutic potential: A consensus statement from the International Atherosclerosis Society (IAS) and the Residual Risk Reduction Initiative (R3i) Foundation. Cardiovasc Diabetol.

[152] Takei K, Nakagawa Y, Wang Y, Han SI, Satoh A, Sekiya M, Matsuzaka T, Shimano H (2017). Effects of K-877, a novel selective PPARα modulator, on small intestine contribute to the amelioration of hyperlipidemia in low-density lipoprotein receptor knockout mice. J Pharmacol Sci.

[153] Fisher FM, Chui PC, Nasser IA, Popov Y, Cunniff JC, Lundasen T, Kharitonenkov A, Schuppan D, Flier JS, Maratos-Flier E (2014). Fibroblast growth factor 21 limits lipotoxicity by promoting hepatic fatty acid activation in mice on methionine and choline-deficient diets. Gastroenterology.

[154] Vu-Dac N, Gervois P, Jakel H, Nowak M, Bauge E, Dehondt H, Staels B, Pennacchio LA, Rubin EM, Fruchart-Najib J (2003). Apolipoprotein A5, a crucial determinant of plasma triglyceride levels, is highly responsive to peroxisome proliferator-activated receptor alpha activators. J Biol Chem.

[155] Ishibashi S, Yamashita S, Arai H, Araki E, Yokote K, Suganami H, Fruchart JC, Kodama T (2016). Effects of K-877, a novel selective PPARα modulator (SPPARMα), in dyslipidaemic patients: A randomized, double blind, active- and placebo-controlled, phase 2 trial. Atherosclerosis.

[156] Arai H, Yamashita S, Yokote K, Araki E, Suganami H, Ishibashi S (2018). Efficacy and safety of pemafibrate versus fenofibrate in patients with high triglyceride and low HDL cholesterol levels: a multicenter, placebo-controlled, double-blind, randomized trial. J Atheroscler Thromb.

[157] Ishibashi S, Arai H, Yokote K, Araki E, Suganami H, Yamashita S (2018). Efficacy and safety of pemafibrate (K-877), a selective peroxisome proliferator-activated receptor α modulator, in patients with dyslipidemia: Results from a 24-week, randomized, double blind, active-controlled, phase 3 trial. J Clin Lipidol.

[158] Ida S, Kaneko R, Murata K (2019). Efficacy and safety of pemafibrate administration in patients with dyslipidemia: a systematic review and meta-analysis. Cardiovasc Diabetol.

[159] Araki E, Yamashita S, Arai H, Yokote K, Satoh J, Inoguchi T, Nakamura J, Maegawa H, Yoshioka N, Tanizawa Y (2019). Efficacy and safety of pemafibrate in people with type 2 diabetes and elevated triglyceride levels: 52-week data from the PROVIDE study. Diabetes Obes Metab.

[160] Yamashita S, Masuda D, Matsuzawa Y (2019). Clinical applications of a novel selective PPARα modulator, pemafibrate, in dyslipidemia and metabolic diseases. J Atheroscler Thromb.

[161] Arai H, Yamashita S, Yokote K, Araki E, Suganami H, Ishibashi S (2017). Efficacy and safety of K-877, a novel selective peroxisome proliferator-activated receptor α modulator (SPPARMα), in combination with statin treatment: Two randomised, double-blind, placebo-controlled clinical trials in patients with dyslipidaemia. Atherosclerosis.

[162] Araki E, Yamashita S, Arai H, Yokote K, Satoh J, Inoguchi T, Nakamura J, Maegawa H, Yoshioka N, Tanizawa Y (2018). Effects of pemafibrate, a novel selective PPARα modulator, on lipid and glucose metabolism in patients with type 2 diabetes and hypertriglyceridemia: a randomized, double-blind, placebo-controlled, phase 3 trial. Diabetes Care.

[163] Yamashita S, Arai H, Yokote K, Araki E, Suganami H, Ishibashi S (2018). Effects of pemafibrate (K-877) on cholesterol efflux capacity and postprandial hyperlipidemia in patients with atherogenic dyslipidemia. J Clin Lipidol.

[164] Benderly M, Graff E, Reicher-Reiss H, Behar S, Brunner D, Goldbourt U (1996). Fibrinogen is a predictor of mortality in coronary heart disease patients. The Bezafibrate Infarction Prevention (BIP) Study Group. Arterioscler Thromb Vasc Biol.

[165] Tofield A (2015). Recurrent atrial fibrillation reduced after renal denervation with pulmonary vein ablation in select patients. Eur Heart J.

[166] Matsuba I, Matsuba R, Ishibashi S, Yamashita S, Arai H, Yokote K, Suganami H, Araki E (2018). Effects of a novel selective peroxisome proliferator-activated receptor-α modulator, pemafibrate, on hepatic and peripheral glucose uptake in patients with hypertriglyceridemia and insulin resistance. J Diabetes Investig.

[167] Pradhan AD, Paynter NP, Everett BM, Glynn RJ, Amarenco P, Elam M, Ginsberg H, Hiatt WR, Ishibashi S, Koenig W (2018). Rationale and design of the Pemafibrate to Reduce Cardiovascular Outcomes by Reducing Triglycerides in Patients with Diabetes (PROMINENT) study. Am Heart J.

[168] Cuthbertson J, Patterson S, O’Harte FP, Bell PM (2009). Investigation of the effect of oral metformin on dipeptidylpeptidase-4 (DPP-4) activity in Type 2 diabetes. Diabet Med.

[169] Bahne E, Sun EWL, Young RL, Hansen M, Sonne DP, Hansen JS, Rohde U, Liou AP, Jackson ML, de Fontgalland D (2018). Metformin-induced glucagon-like peptide-1 secretion contributes to the actions of metformin in type 2 diabetes. JCI Insight..

[170] Maida A, Lamont BJ, Cao X, Drucker DJ (2011). Metformin regulates the incretin receptor axis via a pathway dependent on peroxisome proliferator-activated receptor-α in mice. Diabetologia.

[171] Takashima M, Ogawa W, Hayashi K, Inoue H, Kinoshita S, Okamoto Y, Sakaue H, Wataoka Y, Emi A, Senga Y (2010). Role of KLF15 in regulation of hepatic gluconeogenesis and metformin action. Diabetes.

[172] Hatazawa Y, Tadaishi M, Nagaike Y, Morita A, Ogawa Y, Ezaki O, Takai-Igarashi T, Kitaura Y, Shimomura Y, Kamei Y (2014). PGC-1α-mediated branched-chain amino acid metabolism in the skeletal muscle. PLoS One.

[173] Kobayashi R, Murakami T, Obayashi M, Nakai N, Jaskiewicz J, Fujiwara Y, Shimomura Y, Harris RA (2002). Clofibric acid stimulates branched-chain amino acid catabolism by three mechanisms. Arch Biochem Biophys.

[174] Zemdegs J, Martin H, Pintana H, Bullich S, Manta S, Marqués MA, Moro C, Layé S, Ducrocq F, Chattipakorn N (2019). Metformin promotes anxiolytic and antidepressant-like responses in insulin-resistant mice by decreasing circulating branched-chain amino acids. J Neurosci.

[175] Lynch CJ, Adams SH (2014). Branched-chain amino acids in metabolic signalling and insulin resistance. Nat Rev Endocrinol.

[176] MacDonald MR, Petrie MC, Hawkins NM, Petrie JR, Fisher M, McKelvie R, Aguilar D, Krum H, McMurray JJ (2008). Diabetes, left ventricular systolic dysfunction, and chronic heart failure. Eur Heart J.

[177] Park EJ, Lim SM, Lee KC, Na DH (2016). Exendins and exendin analogs for diabetic therapy: a patent review (2012–2015). Expert Opin Ther Pat.

[178] Younce CW, Burmeister MA, Ayala JE (2013). Exendin-4 attenuates high glucose-induced cardiomyocyte apoptosis via inhibition of endoplasmic reticulum stress and activation of SERCA2a. Am J Physiol Cell Physiol.

[179] Mamas MA, Deaton C, Rutter MK, Yuille M, Williams SG, Ray SG, New J, Gibson JM, Neyses L (2010). Impaired glucose tolerance and insulin resistance in heart failure: underrecognized and undertreated?. J Card Fail.

[180] Doehner W, Frenneaux M, Anker SD (2014). Metabolic impairment in heart failure: the myocardial and systemic perspective. J Am Coll Cardiol.

[181] Antza C, Nirantharakumar K, Doundoulakis I, Tahrani AA, Toulis KA (2019). The development of an oral GLP-1 receptor agonist for the management of type 2 diabetes: evidence to date. Drug Des Devel Ther.

[182] Ban K, Noyan-Ashraf MH, Hoefer J, Bolz SS, Drucker DJ, Husain M (2008). Cardioprotective and vasodilatory actions of glucagon-like peptide 1 receptor are mediated through both glucagon-like peptide 1 receptor-dependent and -independent pathways. Circulation.

[183] Marso SP, Daniels GH, Brown-Frandsen K, Kristensen P, Mann JF, Nauck MA, Nissen SE, Pocock S, Poulter NR, Ravn LS (2016). Liraglutide and cardiovascular outcomes in type 2 diabetes. N Engl J Med.

[184] Pfeffer MA, Claggett B, Diaz R, Dickstein K, Gerstein HC, Køber LV, Lawson FC, Ping L, Wei X, Lewis EF (2015). Lixisenatide in patients with type 2 diabetes and acute coronary syndrome. N Engl J Med.

[185] Holman RR, Bethel MA, Mentz RJ, Thompson VP, Lokhnygina Y, Buse JB, Chan JC, Choi J, Gustavson SM, Iqbal N (2017). Effects of once-weekly exenatide on cardiovascular outcomes in type 2 diabetes. N Engl J Med.

[186] Marso SP, Bain SC, Consoli A, Eliaschewitz FG, Jódar E, Leiter LA, Lingvay I, Rosenstock J, Seufert J, Warren ML (2016). Semaglutide and cardiovascular outcomes in patients with type 2 diabetes. N Engl J Med.

[187] Zelniker TA, Wiviott SD, Raz I, Im K, Goodrich EL, Bonaca MP, Mosenzon O, Kato ET, Cahn A, Furtado RHM (2019). SGLT2 inhibitors for primary and secondary prevention of cardiovascular and renal outcomes in type 2 diabetes: a systematic review and meta-analysis of cardiovascular outcome trials. Lancet.

[188] Zinman B, Wanner C, Lachin JM, Fitchett D, Bluhmki E, Hantel S, Mattheus M, Devins T, Johansen OE, Woerle HJ (2015). Empagliflozin, cardiovascular outcomes, and mortality in type 2 diabetes. N Engl J Med.

[189] Neal B, Perkovic V, Mahaffey KW, de Zeeuw D, Fulcher G, Erondu N, Shaw W, Law G, Desai M, Matthews DR (2017). Canagliflozin and cardiovascular and renal events in type 2 diabetes. N Engl J Med.

[190] Mahaffey KW, Neal B, Perkovic V, de Zeeuw D, Fulcher G, Erondu N, Shaw W, Fabbrini E, Sun T, Li Q (2018). Canagliflozin for primary and secondary prevention of cardiovascular events: results from the CANVAS Program (Canagliflozin Cardiovascular Assessment Study). Circulation.

[191] Uthman L, Baartscheer A, Bleijlevens B, Schumacher CA, Fiolet JWT, Koeman A, Jancev M, Hollmann MW, Weber NC, Coronel R (2018). Class effects of SGLT2 inhibitors in mouse cardiomyocytes and hearts: inhibition of Na(+)/H(+) exchanger, lowering of cytosolic Na(+) and vasodilation. Diabetologia.

[192] Packer M (2017). Activation and inhibition of sodium-hydrogen exchanger is a mechanism that links the pathophysiology and treatment of diabetes mellitus with that of heart failure. Circulation.

[193] Zelniker TA, Wiviott SD, Raz I, Im K, Goodrich EL, Furtado RHM, Bonaca MP, Mosenzon O, Kato ET, Cahn A (2019). Comparison of the effects of glucagon-like peptide receptor agonists and sodium-glucose cotransporter 2 inhibitors for prevention of major adverse cardiovascular and renal outcomes in type 2 diabetes mellitus. Circulation.

